# The Mechanism and Role of *N*^6^-Methyladenosine (m^6^A) Modification in Atherosclerosis and Atherosclerotic Diseases

**DOI:** 10.3390/jcdd9110367

**Published:** 2022-10-25

**Authors:** Quandan Tan, Song He, Xinyi Leng, Danni Zheng, Fengkai Mao, Junli Hao, Kejie Chen, Haisong Jiang, Yapeng Lin, Jie Yang

**Affiliations:** 1 Department of Neurology, The First Affiliated Hospital of Chengdu Medical College, Chengdu 610072, China; 2 Department of Medicine & Therapeutics, The Chinese University of Hong Kong, Hong Kong 999077, China; 3 Biomedical Informatics and Digital Health, School of Medical Sciences, University of Sydney, Sydney NSW 2050, Australia; 4 School of Biomedical Sciences and Technology, Chengdu Medical College, Chengdu 610072, China; 5 School of Public Health, Chengdu Medical College, Chengdu 610072, China; 6 Department of Neurology, Sichuan Provincial People’s Hospital, University of Electronic Science and Technology of China, Chengdu 610072, China; 7 International Clinical Research Center, Chengdu Medical College, Chengdu 610072, China

**Keywords:** *N*^6^-methyladenosine (m^6^A), atherosclerosis, atherosclerotic diseases, diagnostic biomarkers, targeted therapeutics

## Abstract

*N*^6^-methyladenosine (m^6^A) modification is a newly discovered regulatory mechanism in eukaryotes. As one of the most common epigenetic mechanisms, m^6^A’s role in the development of atherosclerosis (AS) and atherosclerotic diseases (AD) has also received increasing attention. Herein, we elucidate the effect of m^6^A on major risk factors for AS, including lipid metabolism disorders, hypertension, and hyperglycemia. We also describe how m^6^A methylation contributes to endothelial cell injury, macrophage response, inflammation, and smooth muscle cell response in AS and AD. Subsequently, we illustrate the m^6^A-mediated aberrant biological role in the pathogenesis of AS and AD, and analyze the levels of m^6^A methylation in peripheral blood or local tissues of AS and AD, which helps to further discuss the diagnostic and therapeutic potential of m^6^A regulation for AS and AD. In summary, studies on m^6^A methylation provide new insights into the pathophysiologic mechanisms of AS and AD, and m^6^A methylation could be a novel diagnostic biomarker and therapeutic target for AS and AD.

## 1. Introduction

Atherosclerosis (AS) is a chronic inflammatory disease with multiple pathological features, such as endothelial dysfunction, vascular inflammation, and cholesterol accumulation. AS can cause artery plaque and stenosis, leading to the occurrence of atherosclerotic diseases (AD), such as coronary artery disease, stroke, and other arterial diseases [1,2]. AD remain the leading causes of death worldwide, and have created a vital global burden, which is still increasing [3,4]. However, the pathogenesis of AS and AD is extremely complex and largely unclear. In a word, it is of great significance to investigate the new mechanism and potential therapeutic targets of AS and AD.

A growing number of studies [5,6] show that post-transcriptional epigenetic modifications are closely related to the processes of AS and AD. *N*^6^-methyladenosine (m^6^A) modifications (one of the common post-transcriptional epigenetic modifications) are involved in the occurrence and development of AS and AD, and are novel and potential therapeutic targets and diagnostic biomarkers for AS and AD [7,8,9].

m^6^A methylation is a post-transcriptional epigenetic modification at the RNA level, which is a process of methylation of adenine at the sixth nitrogen atom catalyzed by RNA methyltransferases. m^6^A methylation is the most prevalent and reversible type of modification in eukaryotic mRNA, and it also plays a role in noncoding RNAs such as microRNAs (miRNAs), long non-coding RNAs (lncRNAs), and circular RNAs (circRNAs) [10,11,12,13]. m^6^A methylation can regulate RNA stability, positioning, transport, splicing, and translation [14,15], which will affect the structure and function of RNAs. It plays a crucial regulatory role in the pathogenesis of various diseases, such as tumors, cardiovascular, and cerebrovascular diseases, etc. [16,17]. Recent studies [7,8,9] have identified the significant role of m^6^A methylation in the occurrence and development of AS and AD. In this review, we describe the relationship between m^6^A and risk factors of AS, highlight its mechanism in the pathogenesis of AS, and elucidate the impact of m^6^A methylation on the development of AS and AD. We also discuss the diagnostic and therapeutic potential of m^6^A methylation regulators for AS and AD. Our review may provide novel insights into the pathophysiologic mechanisms, diagnostic biomarkers, and therapeutic targets for AS and AD.

## 2. Regulators of m^6^A Methylation

**Figure 1:**
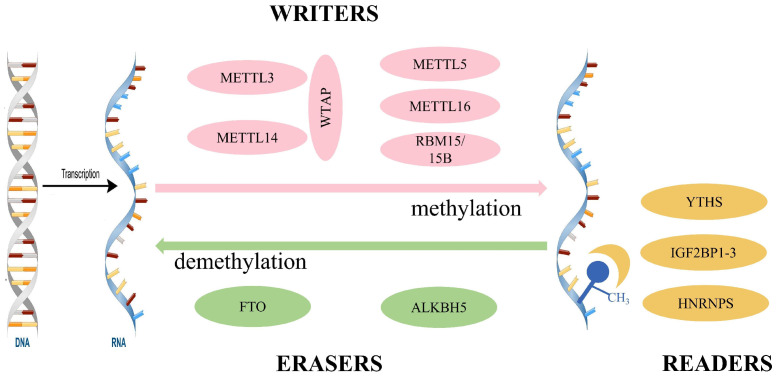
Regulators of m^6^A methylation. MTTL3, methyltransferase-like 3; WTAP, Wilms tumor 1-associated protein; RBM15/15B, RNA binding motif protein 15/15B; FTO, fat mass and obesity-related protein; ALKBH5, alkylation repair homologous protein 5; YTHS, YTDF homeodomain family proteins; IGF2BP1-3, insulin-like growth factor 2 mRNA binding protein; HNRNPS, heterogeneous ribonucleoproteins.

Since its first discovery in the 1970s [18], m^6^A has been identified as the most common mRNA internal modification in most eukaryotic species [18,19,20,21,22,23]. Similar to DNA methylation and histone modification, RNA methylation is a dynamic and reversible modification that regulates gene expression. m^6^A modifications are mediated by three regulators: “writers” (methyltransferases), “erasers” (demethylases), and “readers” (m^6^A-binding proteins) (Figure 1). The m^6^A methylation site has a typical consensus sequence RRACH (R=G or A; H=A, C, or U), which is enriched in the coding sequence and 3′ untranslated region, particularly around stop codon regions [20,21]. Interactions among m^6^A-modified writers, readers, and erasers are involved in the regulation of the RNA life cycle, thereby affecting many physiological and pathological processes, such as cell differentiation, self-renewal, and apoptosis, and the development of cancer, cardiovascular, and metabolic diseases.

### 2.1. Writers

The methyltransferases, also known as the codons or writers, are involved in the composition of the methyltransferase complex (MTC). MTC is composed of methyltransferase-like 3 (METTL3), METTL14, and other related regulators such as Wilms tumor 1-associated protein (WTAP), METTL5, METTL16, and RNA binding motif protein 15/15B (RBM15/15B) [24,25,26,27,28].

METTL3 was discovered in 1997 and it contains two S-adenosylmethionine binding sites called catalytically active methyltransferase domains [29]. METTL14, a homolog of METTL3, plays an important role in structurally supporting RNA binding by providing an RNA-binding scaffold [30,31]. WTAP can bind to the METTL3-METTL14 complex and plays an important role in regulating the localization of the METTL3-METTL14 complex to nuclear foci [32]. In addition, METTL5, METTL16, RBM15/15B, and ZC3H13 play integral roles in m^6^A methylation [25,26,27,28].

### 2.2. Erasers

In contrast to MTC, demethylases are called erasers. Their role is to remove m^6^A methylation [33]. Demethylases include fat mass and obesity-related protein (FTO) and alkylation repair homologous protein 5 (ALKBH5) [22,34]. Although these two enzymes have similar functions, they play different roles in the process of demethylation.

In 2007, Frayling et al. [30] discovered a genetic variation in a gene associated with obesity risk, which was officially named FTO. FTO is abundant in the brain, especially in neurons. Hence, it may play an important role in the brain. FTO-dependent m^6^A demethylation contributes to human obesity and regulates energy balance, which is critical for its biological role in the cardiovascular system [35]. ALKBH5 is another nuclear-localized m^6^A demethylase. Zheng et al. [22] found that m^6^A total RNA levels were reduced in ALKBH5-overexpressing cells, which have been shown to regulate mRNA export, RNA metabolism, and mRNA assembly in nuclear speckles. In addition, ALKBH5 also plays a key role in biological processes such as cell cycle, stress response, and apoptosis [36].

### 2.3. Readers

Similar to DNA methylation, the biological function of m^6^A methylation is mediated by the recognition of m^6^A sites by m^6^A “readers”. m^6^A readers include YTDF homeodomain family proteins (YTDF1, YTDF2, YTDF3, YTDC1, and YTDC2), insulin-like growth factor 2 mRNA binding protein (IGF2BP1-3), and heterogeneous ribonucleoproteins (HNRNPA2B1, HNRNPC, and HNRNPG) [37].

Different readers have different biological functions. YTHDF1 promotes the translation of m^6^A methylated mRNAs, YTHDF2 accelerates the decay of m^6^A methylated mRNAs, and YTHDF3, together with YTHDF1 and YTHDF 2, significantly enhances the metabolism of m^6^A methylated mRNAs in the cytoplasm [38]. In addition, IGF2BP expressed in the cytoplasm not only enhances mRNA stability but also increases translation efficiency [39]. Moreover, HNRNPG is involved in mRNA splicing and regulates pre-mRNA processing [40].

## 3. The Effects of m^6^A Methylation in AS Major Risk Factors

**Figure 2:**
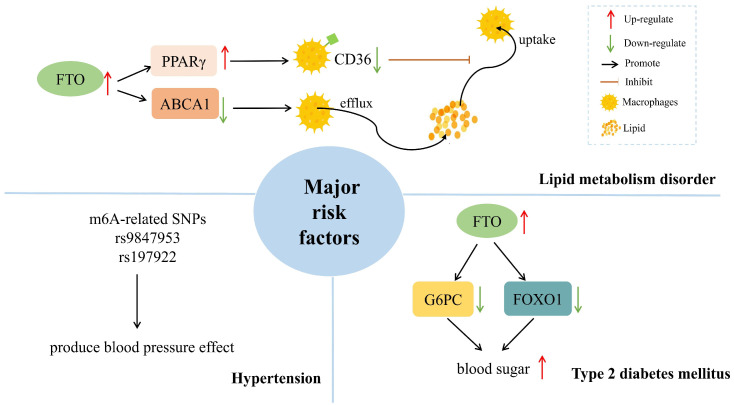
Effects of m^6^A methylation on the development of AS major risk factors. PPARγ, proliferator-activated receptor γ; SNPs, single nucleotide polymorphisms; G6PC, glucose-6-phosphatase catalytic subunit; FOXO1; forkhead box protein O1.

Dyslipidemia, hypertension, and diabetes are the most common risk factors for AS. We reviewed the effects of m^6^A methylation on the development of AS major risk factors as follows (Figure 2).

### 3.1. Lipid Metabolism Disorder

Atherosclerotic lesions are based on lipid metabolism disorders [41]. Serum low-density lipoprotein levels are negatively correlated with m^6^A levels [42]. In addition, FTO catalyzes the demethylation of m^6^A methylation to alter mRNA processing, maturation, and translation of lipid-related genes [22,43,44].

Previous studies [45] have demonstrated that FTO inhibits the macrophage uptake of extracellular lipids, promotes intracellular lipid efflux, and inhibits macrophage lipid accumulation and foam cell formation. Scavenger receptor CD36, the primary transporter mediating extracellular lipid uptake by macrophages, is directly targeted by the oxisome proliferator-activated receptor γ (PPARγ). Mo et al. [46] subsequently observed that FTO-dependent m^6^A demethylation reduced PPARγ protein expression, resulting in downregulation of CD36 expression and decreased lipid uptake in RAW264.7 cells. Furthermore, Wu et al. showed that [47] FTO promotes AMPK phosphorylation and up-regulated ATP-binding cassette transporter A1 (ABCA1) in macrophages of mice. ABCA1 consumes ATP to mediate intracellular cholesterol efflux, which strongly prevents excessive lipid accumulation in macrophages. Reduced AMPK activity was shown to block FTO-induced upregulation of ABCA1. Additionally, FTO increases ABCA1 expression in an AMPK activity-dependent manner [46,48].

In conclusion, FTO is the key to regulating lipid homeostasis. m^6^A demethylation of FTO inhibits macrophage lipid influx by downregulating PPARγ protein expression and accelerates cholesterol efflux by phosphorylating AMPK, thereby preventing foam cell formation and development of AS.

### 3.2. Hypertension

Hypertension is one of the main risk factors for AS, but the mechanism by which hypertension promotes the occurrence of AS is unclear. However, one study showed [49] that epigenetics could influence the pathogenesis of hypertension.

Wu et al. [50] demonstrated by m^6^A high-throughput sequencing analysis that the average abundance of m^6^A was reduced in microvascular pericytes of spontaneously hypertensive rats. This means that m^6^A methylation may regulate hypertension in mammals. Genetic variation affects m^6^A expression by altering the RNA sequence of the target site, which can be referred to as m^6^A-related single nucleotide polymorphisms (SNPs) [51]. The study by Mo et al. [52] showed that many m^6^A-related SNPs, such as rs9847953 and rs197922, affect the expression of related genes, such as C1orf167, DOT1L, and thus produce blood pressure effects. These findings may shed light on the underlying mechanism of hypertension from the perspective of m^6^A modification.

### 3.3. Type 2 Diabetes Mellitus (T2DM)

T2DM is a common risk factor for AS. A previous study [53] showed that specific variants in FTO could predispose individuals to T2DM. Among these variants, the FTO rs9939609 (T > A) polymorphism is the most studied; for example, in the Oulu Project Elucidation of Atherosclerosis Risk study [54], it was shown that the FTO rs9939609 minor allele individuals with genetic variants had significantly higher rates of cardiovascular disease events or deaths. Yang et al. [55] showed that FTO positively regulates gluconeogenesis-related genes, such as the glucose-6-phosphatase catalytic subunit (G6PC) and forkhead box protein O1 (FOXO1), in an m^6^A-dependent manner. In T2DM patients, decreased m^6^A promotes hepatic gluconeogenesis, which leads to increased blood glucose by reducing the expression of gluconeogenesis-related genes. Furthermore, in a study of pancreatic islet cells from T2DM patients [56], m^6^A methylation was significantly reduced in β cells, but not in α cells, providing evidence that m^6^A methylation controls cellular insulin secretion. This evidence suggests that m^6^A methylation plays a vital role in T2DM.

## 4. The Mechanisms of m^6^A Methylation in AS

**Figure 3:**
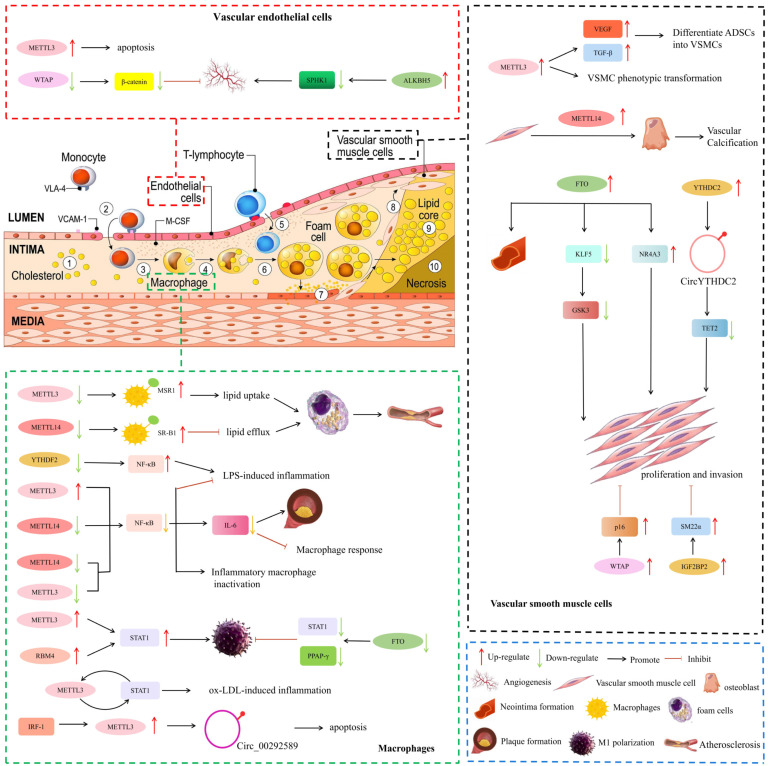
Potential mechanisms involved in m^6^A methylation-mediated regulation of AS. NR4A3, nuclear receptor subfamily 4 group A member 3; GSK3, glycogen synthase kinase 3; TET2, ten-eleven translocation 2; SM22α, smooth muscle 22α; ADSCs, adipose-derived stem cells; VSMCs, Vascular Smooth Muscle Cells; MSR1, macrophage scavenger receptor A; SR-B1, scavenger receptor B type 1; STAT1, signal transducer and activator of transcription 1; IRF-1, interferon regulatory factor-1.

Endothelial cells, macrophages, and smooth muscle cells are the most important initiating and developing cell types of AS. We summarized the mechanism by which m^6^A regulates these cell types to induce AS as follows (Figure 3).

### 4.1. Vascular Endothelial Cells

During the initial stages of AS, endothelial dysfunction and morphological damage occur, which lead to leukocyte adhesion, vasoconstriction, platelet aggregation, and thrombosis [57]. Vascular endothelial cell dysfunction is a key factor in the pathogenesis of AS.

m^6^A methylation plays a major role in post-transcriptional regulation in vascular endothelial cells. Zhu et al. [58] found that human cytomegalovirus (HCMV) infection could induce abnormally elevated m^6^A methylation, especially METTL3 and YTHDF3, leading to endothelial cell apoptosis. Wang et al. [59] used RNA transcriptome sequencing and found that in cerebral arteriovenous malformations, the expression levels of WTAP were significantly reduced but could inhibit endothelial cell angiogenesis.

m^6^A demethylation also plays important roles in endothelial cell angiogenesis. For example, Rajesh Kumari et al. [60] found that ALKBH5 levels were up-regulated after ischemia and correlated with the maintenance of ischemia-induced endothelial cell angiogenesis. ALKBH5 contributes to the maintenance of endothelial angiogenesis after acute ischemic stress by reducing SPHK1 m^6^A methylation and downstream eNOS-AKT signaling.

### 4.2. Macrophages Response and Inflammation

m^6^A methylation can influence AS progression by affecting macrophage cholesterol efflux and cell death. Cholesterol accumulation in macrophages, foam cell formation, and atherosclerotic lesions are all affected by macrophage cholesterol efflux capacity [61]. Zhao et al. [62] showed that during AS, oxidized low-density lipoprotein (ox-LDL) induced the expression of dead box protein 5 (DDX5) in macrophages and restricted the METTL3 function. METTL3 can transfer methyl groups to macrophage scavenger receptor A (MSR1) mRNA. Eventually, MSR1 mRNA stabilizes, and more MSR1 is synthesized. The uptake of more lipids further promotes the formation of foam cells, leading to the progression of AS. However, the specific mechanism of METTL3 inhibition by DDX5 is unclear. Park et al. [63] showed that MELL14 knockout attenuated cholesterol efflux and promoted foam cell formation by affecting m^6^A levels of scavenger receptor B type 1 (SR-B1) mRNA.

Inflammation is one of the major and fundamental pathological processes for all stages of AS [64]. The transformation of macrophages into an inflammatory phenotype is closely related to the progression of AS. Signal transducer and activator of transcription 1 (STAT1) is a key transcription factor whose activation leads to signaling cascades activated by pro-inflammatory macrophages. Liu et al. [65] showed that METTL3 has been shown to directly methylate STAT1 mRNA to increase mRNA stability, thereby upregulating STAT1 expression and promoting the polarization of M1 macrophages. Huang et al. [66] demonstrated that RBM4 regulates M1 macrophage polarization by targeting STAT1-mediated glycolysis. This study shows that RBM4 may be a candidate for regulating M1 polarization and inflammatory responses in macrophages. In addition, Gu et al. [67] found that the FTO gene knockout of m^6^A demethylase inhibited the phosphorylation of key proteins in the NF-κB signaling pathway, and was involved in reducing the mRNA stability of STAT1 and PPARγ through YTHDF2, thereby hindering macrophages polarization of cells. Similarly, Li et al. [68] also showed that ox-LDL stimulation significantly increased m^6^A-modified mRNA levels in macrophages. METTL3 promotes ox-LDL-triggered inflammation by interacting with STAT1 protein and mRNA in macrophages. In summary, m^6^A via the STAT1 pathway plays an important role in macrophage inflammation in AS.

In addition, m^6^A via the NF/κB signaling pathway also plays a crucial role in macrophage inflammation in AS. Wang et al. [69] showed that METTL3 reduced lipopolysaccharide (LPS)-induced macrophage inflammatory response by inhibiting the NF-κB pathway. However, Yu et al. [70] showed that downregulation of YTHDF2 significantly increased the LPS-induced expression of pro-inflammatory cytokines, such as IL-6 and TNF-α, and activated the MAPK and NF-κB signaling pathways. In addition, Yu et al. [71] found that the inhibition of METTL14 and METTL3 expression in macrophages could abolish m^6^A methylation of NF-κB mRNA, affect the stability of NF-κB mRNA, and ultimately lead to the inactivation of inflammatory macrophages, thereby significantly alleviating the progression of AS. Zheng et al. [72] found that Mettl14 knockout significantly reduced macrophage inflammatory response and atherosclerotic plaque formation through the NF-κB/IL-6 signaling pathway.

Moreover, Zhang et al. [73] identified a role for METTL3 in promoting oxidized LDL-induced monocyte inflammation. Guo et al. [74] showed that overexpression of interferon regulatory factor-1 (IRF-1) promoted apoptosis and inflammatory responses in atherosclerotic macrophages by upregulating m^6^A methylation levels and METTL3 expression on circ_0029589.

### 4.3. Vascular Smooth Muscle Cell (VSMC)

During AS progression, contractile VSMCs undergo phenotypic transformation into proliferative synthetic cells that generate an extracellular matrix, form fibrous caps, and stabilize plaques [75]. Accumulating evidence suggests that m^6^A can affect the pathophysiological function of VSMCs.

The “writers” of m^6^A methylation are involved in VSMC proliferation and migration. Lin et al. [76] showed that hypoxia can affect METTL3 expression and further affect m^6^A modification of related factors such as VEGF and TGF-β, thereby inducing adipose-derived stem cells (ADSCs) to differentiate into VSMCs. Chen et al. [77] found that overexpressed METTL14 increased m^6^A methylation by promoting the transformation of VSMCs to osteoblasts, and played an important role in the pathological mechanism of vascular calcification. Furthermore, in the study by Zhu et al. [78], the expression of WTAP in VSMCs altered cell proliferation and migration. Total notoginseng saponins regulate p16 m^6^A methylation by promoting WTAP expression, thereby inhibiting intimal thickening.

The “erasers” of m^6^A demethylases have been reported to promote VSMC proliferation and migration. For example, Ma et al. [79] demonstrated that both FTO overexpression and Ang II-induced FTO expression promoted VSMC proliferation and migration. FTO promotes the expression of Kruppel-like factor 5 (KLF5) mRNA by reducing the m^6^A methylation of KLF5 mRNA, thereby upregulating the expression of downstream glycogen synthase kinase 3 (GSK3). Similarly, Huo et al. [80] also showed that FTO promoted Ang II-induced VSMC proliferation and inflammatory response by demethylating the m^6^A methylation of nuclear receptor subfamily 4 group A member 3 (NR4A3) mRNA. In addition, Deng et al. [81] established a rat carotid artery balloon injury model to confirm the role of the FTO in neointima formation.

Different m^6^A “readers” function differently in VSMC proliferation and migration. Yuan et al. [82] found that YTHDC2-mediated m^6^A modification stabilizes circYTHDC2, which promotes VSMC proliferation and migration by negatively regulating the expression of ten-eleven translocation 2 (TET2). However, Zhang et al. [83] showed that IGF2BP2 increased the stability of smooth muscle 22α (SM22α) mRNA by acting as a “reader” for m^6^A-modified SM22α, inhibited the proliferation and migration of VSMC, and inhibited intimal hyperplasia.

## 5. The Role of m^6^A Methylation in AS and AD

m^6^A methylation not only causes the most common atherosclerotic diseases (such as coronary heart disease (CHD) and ischemic stroke (IS) through AS. Moreover, m^6^A also plays an important role in the injury and repair of CHD and IS. In Table 1, we reviewed the progress of m^6^A methylation regulator-guided epigenetic modification in AS and AD.

### 5.1. AS

AS is the main cause of CHD and IS [99]. Quiles Jiménez et al. [6] used mass spectrometry to analyze m^6^A methylation levels in tissue from non-atherosclerotic arterial and carotid atherosclerotic patients, which showed the changes in the expression levels of m^6^A writers, erasers, and readers in atherosclerotic tissue. The findings of Wu et al. [42] showed that m^6^A methylation levels were significantly reduced in peripheral blood leukocytes of atherosclerotic patients and mice. The bioinformatic analysis indicated that differentially methylated genes were involved in the pathogenesis of AS. These findings suggest that m^6^A methylation is involved in the occurrence and progression of AS.

METTL3-dependent m^6^A methylation was recently shown to play an important role in AS. For example, Yao et al. [84] demonstrated that METTL3 promotes the translation of low-density lipoprotein receptor-related protein 6 (LRP6) and dishevelled 1 (DVL1) in human umbilical vein endothelial cells (HUVEC) under hypoxic stress in a YTHDF1-dependent manner, thereby exerting an angiogenesis effect. Zhang et al. [73] demonstrated that METTL3 plays a role in ox-LDL-induced monocyte inflammation, in which METTL3 and YTHDF2 synergistically modify PGC-1α mRNA, mediate its degradation, and reduce PGC-1α protein levels, thereby enhancing the inflammatory response. This study provides new insights into the role of METTL3-dependent m^6^A methylation of PGC-1α mRNA in the inflammatory response of monocytes. In addition, Dong et al. [85] explored the role and molecular mechanism of m^6^A-METTL3 in AS progression from an in vivo perspective using an AS mouse model and an in vivo chick embryo chorioallantoic membrane assay. The results indicated that METTL3 knockout prevented AS progression through IGF2BP1 inhibition of the JAK2/STAT3 pathway. Furthermore, Chien et al. [86] showed that METTL3 up-regulated NOD-like receptor protein 1 (NLRP1) and down-regulated Kruppel-like factor 4 (KLF4) in an m^6^A-dependent manner. METTL3 exerts pro-inflammatory effects in HUVEC or mouse aortic endothelial cells exposed to pro-atherosclerotic oscillatory stress or TNF-α stimulation, thereby promoting inflammatory cell adhesion and AS pathogenesis. In a recent study, Chen et al. [87] found that silencing METTL3 alleviated AS progression in mice. Silencing METTL3 suppressed m^6^A levels and decreased the binding of DGCR8 to pri-miR-375, further limiting the expression of miR-375-3p. miR-375-3p targets PDK1 transcription. Ultimately silencing METTL3 plays a role in stabilizing AS plaques. However, Li et al. [88] reported a protective role of METTL3 in AS. The authors found that METTL3 promotes m^6^A-dependent degradation of epidermal growth factor receptor (EGFR) mRNA, a molecule associated with vascular endothelial cell (EC) dysfunction, thereby attenuating the progression of endothelial atherosclerosis.

Similarly, METTL14 also plays a pivotal role in the process of AS. Jian et al. [89] constructed a model of EC inflammation induced by TNF-α. With an increase in the expression of METTL14 in endothelial cells stimulated with TNF-α, METTL14 increases the m^6^A methylation of FOXO1, promoting its expression, which triggers endothelial inflammatory responses and the development of AS. Subsequent in vivo experiments showed that METTL14 knockout could inhibit AS plaque development in an m^6^A-dependent manner in METTL14 knockout mice. Furthermore, Zhang et al. [90] pointed out that METTL14 promoted the production of mature miR-19a by increasing the expression of m^6^A in miR-19a, thereby accelerating the invasion and proliferation of cardiovascular ECs. Chen et al. [100] showed that the m^6^A methylation of Zinc finger NFX type 1 (ZNFX1) antisense RNA 1 (ZFAS1) was significantly higher in AS patients than in controls, and that m^6^A methylation in ZFAS1 was regulated by METTl14. Tang et al. [91] found that METTl14 affects the expression of downstream ADAM10/RAB22A by affecting the m^6^A methylation of LncRNA ZFAS1, thereby participating in cholesterol metabolism and vascular inflammation, and ultimately regulating the occurrence and development of AS. Liu et al. [92] demonstrated that silencing METTL14 attenuates the development of AS through the m^6^A methylation of p65 mRNA by establishing an in vitro atherosclerotic cell model and an in vivo high-fat diet mouse model. Zheng et al. [72] showed that Mettl14 plays a crucial role in macrophage inflammation in AS through the NF-κB/IL-6 signaling pathway. METTL14 knockout significantly reduced the macrophage inflammatory response and atherosclerotic plaque formation.

In addition, demethylases also play an important role in AS. For example, Deng et al. [81] established a rat carotid artery balloon injury model, which confirmed that FTO could induce neointima formation. Huo et al. [80] used Ang II to construct vascular smooth muscle cells (VSMC) and vascular inflammation models in vitro and in vivo, and confirmed that the FTO/NR4A3 axis plays a key role in Ang II-induced VSMC proliferation and inflammation. Wu et al. [42] found that ox-LDL-induced ALKBH1 promotes myocardial infarction-associated transcript (MIAT) transcription by promoting the binding of hypoxia-inducible factor 1α (HIF1α). In addition, ALKBH1 knockdown inhibited ox-LDL-induced MIAT expression. All in all, the present findings suggest that the ALKBH1-m^6^A axis may control atherosclerotic plaque progression by regulating MIAT expression.

### 5.2. AD

#### 5.2.1. CHD

CHD, also known as ischemic cardiomyopathy, refers to the clinical syndrome of long-term myocardial ischemia caused by coronary atherosclerosis, resulting in diffuse myocardial fibrosis [101].

Mathiyalagan et al. [94] demonstrated for the first time that mRNA m^6^A methylation was significantly higher in ischemic myocardium than in non-ischemic regions. Song et al. [8] demonstrated that m^6^A RNA methylation is involved in the development of myocardial hypoxia/reperfusion injury by regulating autophagy. Deng et al. [81] identified differentially methylated m^6^A sites in mRNAs and lncRNAs between peripheral blood mononuclear cells of the CHD group and control group. These studies suggest that m^6^A RNA methylation plays a crucial role in CHD.

Song et al. [8] established a mice model of hypoxia-reperfusion and ischemia-reperfusion and found that the m^6^A methylation levels in mice cardiomyocytes increased, and METTL3 is the main cause of abnormal modification of m^6^A methylation. Silencing METTL3 enhances autophagic flux and inhibits cardiomyocyte apoptosis in hypoxic/reoxygenated cardiomyocytes. However, the overexpression of METTL3 or inhibition of m^6^A demethylase ALKBH5 promoted cardiomyocyte apoptosis. This suggests that METTL3 is a negative regulator of autophagy. Similarly, WTAP promotes endoplasmic reticulum (ER) stress and apoptosis by increasing mRNA m^6^A levels of activated transcription factor 4 (ATF4), a transcription factor that controls the expression of ER-related genes, and up-regulates its expression, thereby aggravating myocardial I/R injury [93].

FTO-mediated m^6^A demethylation is also associated with myocardial I/R injury. FTO can selectively demethylate sarcoplasmic reticulum Ca^2+^-ATPase (SERCA2A), myosin heavy chain 6/7 (MYH6/7), ryanodine receptor 2 (RYR2), and other mRNAs that affect cardiac calcium homeostasis, myofibril synthesis, and contractile function. It increases the transcription and translation of the above genes through an m^6^A-dependent pathway, thereby reversing ischemic injury [94]. Myosin heavy chain-related RNA transcript (MHRT) is a heart-specific lncRNA derived from the antisense strand of the MYH7 gene. Shen et al. [95] demonstrated that overexpression of FTO inhibited apoptosis in I/R-treated cardiomyocytes by reducing the m^6^A modification of MHRT.

In addition, overexpression of ALKBH5 can reverse the damaging effects of METTL3 on cardiomyocytes [8]. Another study explored the effect and mechanism of ALKBH5 on angiogenesis after ischemia. Zhao et al. [96] demonstrated that ALKBH5 negatively regulates angiogenesis after ischemia by reducing the mA levels of WNT family member 5A (WNT5A) mRNA and by promoting its degradation in cardiac microvascular endothelial cells.

#### 5.2.2. IS

The stenosis of the cerebral arterial lumen often occurs due to AS, resulting in ensuing thrombosis and IS. Emerging evidence [9,102] suggests that m^6^A methylation is involved in the injury and repair of IS.

Si et al. [97] established an oxygen–glucose deprivation/reperfusion model in primary cortical neurons and PC12 cells by using a middle cerebral artery occlusion model in rats to explore m^6^A methylation of potential mechanisms involved in stress granule (SG) formation in the early stages of acute ischemic stroke. Both in vitro and in vivo results showed that METTL3 protein, m^6^A levels, and miR-335 expression were significantly decreased with prolonged reperfusion time. The finding suggests that METTL3-mediated m^6^A methylation plays an important role in promoting SG formation and reducing IS damage in the early stages of disease.

Similar to METTL3, YTHDC1 also plays a protective role in the pathological process of IS. Zhang et al. [98] found that the knockout of YTHDC1 aggravated ischemic brain injury, while the overexpression of YTHDC1 protected rats from brain injury; mechanistically, YTHDC1 promotes PTEN mRNA degradation to increase Akt phosphorylation, thereby promoting neuronal survival, especially after ischemia.

## 6. Potential Diagnostic Biomarkers and Therapeutic Targets of m^6^A for AS and AD

The above findings provide new information for understanding the molecular pathogenesis of AS and exploring potential diagnostic biomarkers and therapeutic targets for AS and AD.

### 6.1. Potential Diagnostic Biomarkers of m^6^A for AS and AD

The levels of m^6^A methylation in the tissue of AS patients are significantly lower than that of non-AS patients and that of early AS patients. The expression levels of WTAP, METTL3, YTHDF2, and FTO are significantly lower than those of non-AS patients [6]. In addition, m^6^A levels in peripheral blood leukocytes are negatively correlated with carotid plaque size and thickness [42]. Thus, these biomarkers may potentially be used in the future for the early diagnosis of AS. In addition, the m^6^A levels in peripheral blood mononuclear cells of CHD patients are significantly lower than that of the control group, and the expression levels of FTO, METTL14, and ALKBH5 in the CHD patients are also lower than those of the control group [81]. Moreover, global m^6^A levels in ipsilateral cortical tissue around cerebral infarction are significantly elevated after transient focal ischemia, and FTO levels are significantly reduced after stroke [9]. Taken together, these findings indicate that m^6^A may be a potential biomarker for the diagnosis of AD. In summary, m^6^A levels in RNA may prove to be a valuable diagnostic biomarker for AS and AD. However, the relationship between m^6^A and AS or AD, and its diagnostic specificity and sensitivity, would need to be confirmed by clinical studies with easy access to specimens such as peripheral blood.

### 6.2. Potential Therapeutic Targets of m^6^A for AS and AD

Numerous studies have shown that methyltransferases are associated with aberrant m^6^A modification and lead to the development of AS and AD, which indicate that m^6^A methylation may be the potential therapeutic target. METTL3 promotes ox-LDL-triggered inflammation by interacting with STAT1 protein and mRNA in macrophages [68]. METTL14-mediated m^6^A modification of ZFAS1/RAB22A may play an important role in AS [100], and METTL14 plays a crucial role in macrophage inflammation in AS through the NF-κB/IL-6 signaling pathway [72]. In addition, experiments in vivo have shown that silencing METTL3 can stabilize atherosclerotic plaques [87]. Moreover, METTL14 knockout can inhibit the development of AS plaques [89], and silencing METTL14 can alleviate the development of AS [92]. These indicated that METTL3 and METTL14 may be promising therapeutic targets for the clinical treatment of AS. Likewise, ALKBH5 can negatively regulate angiogenesis after ischemia [96]. Thus, targeting ALKBH5 may be a potential therapeutic option for CHD. Additionally, a key link between METTL3-ALKBH5 and autophagy provides a new direction for m^6^A methylation therapy in CHD [8]. Moreover, in IS, METTL3-mediated m^6^A modification plays an important role in reducing damage in the early stages of stroke [97]. Furthermore, YTHDC1 is a novel regulator of neuronal survival [98]. However, further experimental and clinical evidence is needed to confirm these potential therapeutic targets.

Based on the above therapeutic targets, exploratory research on the treatment of AS with chemical drugs and botanical drugs are ongoing. In terms of chemical drugs, Zhu et al. [58] showed that vitamin D3 inhibited HCMV-induced vascular endothelial cell apoptosis by correcting m^6^A modification of mitochondrial calcium transporter mRNA, which was regulated by METTL3 and YTHDF3. This study highlighted the significance of vitamin D3 supplementation in HCMV-induced AS. For botanical drugs, several studies have also shown that Chinese herbal medicine could significantly delay the onset and progression of AS [71,103,104]. Hua Tuo Zai Zao Wan (HTZZW) was the most frequently studied traditional Chinese medicine. In the study by Yu et al. [71], positive effects were observed in AS mice treated with HTZZW. HTZZW exerts its effect through epigenetic regulation, which can regulate the expression of METTL14 and METTL3 in macrophages, thereby eliminating the m^6^A modification of NF-κB mRNA, and finally leading to the inactivation of macrophages, which has the effect of preventing AS.

In conclusion, m^6^A modifications are potential therapeutic targets for AS and AD, and drugs that affect m^6^A methylation are expected to be explored in AS and AD treatment in the future.

## 7. Discussion and Perspectives

This review expounds on the impact of m^6^A methylation on the main risk factors for AS, such as lipid metabolism disorders, hypertension, and hyperglycemia. We also describe the m^6^A methylation mechanisms that may contribute to the development of AS, including vascular endothelial cell injury, macrophage responses, inflammation, and the proliferation and migration of smooth muscle cells. We then summarized the pathophysiological role of m^6^A methylation in AS and AD, and discussed m^6^A methylation and its regulators as diagnostic biomarkers and treatment targets.

In terms of mechanism, the study of m^6^A revealed a potential link between this epigenetic modification and AS and AD. However, the mechanism of AS is very complex, and the specific association between m^6^A methylation and AS remains to be elucidated. Studies of m^6^A methylation in AS have mainly focused on METTL3 and METTL14 expression. Future studies should also explore how other m^6^A regulators, such as erasers and readers, regulate the expression of downstream proteins, and the interactions between m^6^A writers, erasers, and readers. In addition, it is unclear whether m^6^A methylation crosstalk with other epigenetics, such as non-coding RNA, DNA methylation, and histone modification, occurs in the development of AS.

In terms of clinical application, m^6^A methylation is dynamic and reversible, and has important implications for the diagnosis, prevention, and treatment of AS and AD. For diagnostic application, most studies on differential expression of m^6^A levels in AS and AD were performed in animal, and most of them are from local tissues (such as artery, heart, or brain), which is difficult to obtain clinically. In addition, a comprehensive study of the relationship between m^6^A methylation from peripheral blood and the disease in human is badly needed, which will help identify potential biomarkers for the diagnosis of AS and AD. All in all, it is a beautiful blueprint to use a simple detection method to determine the level of m^6^A or related proteins in peripheral blood to achieve the purpose of diagnosing AS and AD. For therapeutic application, m^6^A inhibitors or agonists for the treatment of AS are still at the stage of animal experiments, and more effective drugs and new therapeutic strategies related to m^6^A remain to be discovered.

## 8. Conclusions

Studies on m^6^A methylation provide new insights into the pathophysiologic mechanisms of AS and AD, and m^6^A methylation will be a novel diagnostic biomarker and therapeutic target for AS and AD in the near future.

## Figures and Tables

**Table 1:** m^6^A methylation regulator-guided epigenetic modification in AS and AD.

Atherosclerotic Process	m^6^A Regulators	Expression	Target Gene	Main Function	Reference
AS	METTL3	↑	LRP6 and DVL1	Enhances translation of LRP6 and DVL1, modulates Wnt signaling, and thus exerts angiogenic effects	[84]
	METTL3	↑	PGC-1α mRNA	Promotes mitochondrial dysfunction and ox-LDL-induced inflammation	[73]
	METTL3	↓	JAK2/STAT3	Alleviates ox-LDL-induced endothelial cell dysfunction, prevents in vivo angiogenesis of developing embryos, and hinders progression in AS mice models	[85]
	METTL3	↑	NLRP1 and KLF4	Up-regulates NLRP1, down-regulates KLF4, hypermethylates m^6^A, and triggers atherosclerotic response	[86]
	METTL3	↑	miR-375-3p/PDK1	Makes AS plaques more vulnerable	[87]
	METTL3	↑	EGFR	Promotes EGFR degradation and alleviates endothelial atherogenic progression	[88]
	METTL14	↑	FOXO1	Increases FOXO1 m^6^A methylation, aggravates endothelial inflammation and AS	[89]
	METTL14	↓	miR-19a	Inhibits the proliferation and invasion of ASVEC	[90]
	METTL14	↑	LncRNA ZFAS1	Plays a vital role in AS	[91]
	METTL14	↓	p65 mRNA	Relieves the development of AS	[92]
	METTL14	↓	NF-κB/IL-6	Reduces the inflammation response of macrophages and the development of AS plaques	[72]
	FTO	↑	Not known	Modulates neointima formation in vivo	[81]
	FTO	↓	NR4A3	Alleviates AngII-induced VSMC proliferation and inflammatory response	[80]
	ALKBH5	↓	HIF1α	Inhibits the expression of MIAT induced by ox-LDL	[42]
CHD	METTL3	↑	TFEB	Promotes cardiomyocyte apoptosis	[8]
	WTAP	↑	ATF4	Promotes endoplasmic reticulum stress and apoptosis, aggravates myocardial I/R injury	[93]
	FTO	↑	SERCA2A MYH6/7 RYR2	Reverses ischemic damage	[94]
	FTO	↑	MHRT	Inhibits cardiomyocyte apoptosis	[95]
	ALKBH5	↓	TFEB	Promotes cardiomyocyte apoptosis	[8]
	ALKBH5	↑	WNT5A	Regulates angiogenesis after ischemia	[96]
IS	METTL3	↑	miR-335	Promotes formation of SG and reduces damage of IS	[97]
	YTHDC1	↑	Not known	Protects rats from brain damage	[98]

↑, high expression; ↓, low expression; AS, atherosclerosis; METTL3, methyltransferase-like 3; LRP6, lipoprotein receptor-related protein 6; DVL1, dishevelled 1; ox-LDL, oxidized low-density lipoprotein; NLRP1, NOD-like receptor protein 1; KLF4, Kruppel-like factor 4; EGFR, epidermal growth factor receptor; FOXO1, forkhead box protein O1; ASVEC, atherosclerotic vascular endothelial cell; LncRNA, long non-coding RNA; ZFAS1, Zinc finger NFX type 1 antisense RNA 1; FTO, fat mass and obesity-related protein; NR4A3, nuclear receptor subfamily 4 group A member 3; ALKBH5, alkylation repair homologous protein 5; HIF1α, hypoxia-inducible factor 1α; CHD, coronary artery heart disease; MIAT, myocardial infarction-associated transcript; TFEB, transcription factor EB; WTAP, Wilms tumor 1-associated protein; ATF4, activated transcription factor 4; SERCA2A, sarcoplasmic reticulum Ca^2+^-ATPase; MYH6/7, myosin heavy chain 6/7; RYR2, ryanodine receptor 2; MHRT, myosin heavy chain-related RNA transcript; WNT5A, WNT family member 5A; IS, ischemic stroke; SG, stress granule; YTHDC1, YTH Domain Containing 1.

## Data Availability

Not applicable.

## References

[1] Rohde L.E., Lee R.T. (2003). Pathophysiology of Atherosclerotic Plaque Development and Rupture: An Overview. Semin. Vasc. Med..

[2] Chen L., Zheng J., Xue Q., Zhao Y. (2019). YKL-40 promotes the progress of atherosclerosis independent of lipid metabolism in apolipoprotein E^−/−^ mice fed a high-fat diet. Heart Vessel..

[3] Björkegren J.L.M., Lusis A.J. (2022). Atherosclerosis: Recent developments. Cell.

[4] Collaborators G.B.D.S. (2021). Global, regional, and national burden of stroke and its risk factors, 1990-2019: A systematic analysis for the Global Burden of Disease Study 2019. Lancet Neurol..

[5] Herrington W., Lacey B., Sherliker P., Armitage J., Lewington S. (2016). Epidemiology of Atherosclerosis and the Potential to Reduce the Global Burden of Atherothrombotic Disease. Circ. Res..

[6] Quiles-Jiménez A., Gregersen I., de Sousa M.M.L., Abbas A., Kong X.Y., Alseth I., Holm S., Dahl T.B., Skagen K., Skjelland M. (2020). *N*^6^-methyladenosine in RNA of atherosclerotic plaques: An epitranscriptomic signature of human carotid atherosclerosis. Biochem. Biophys. Res. Commun..

[7] Liu M., Xu K., Saaoud F., Shao Y., Zhang R., Lu Y., Sun Y., Drummer C., Li L., Wu S. (2022). 29 m^6^A-RNA Methylation (Epitranscriptomic) Regulators Are Regulated in 41 Diseases including Atherosclerosis and Tumors Potentially via ROS Regulation—102 Transcriptomic Dataset Analyses. J. Immunol. Res..

[8] Song H., Feng X., Zhang H., Luo Y., Huang J., Lin M., Jin J., Ding X., Wu S., Huang H. (2019). METTL3 and ALKBH5 oppositely regulate m^6^A modification of *TFEB* mRNA, which dictates the fate of hypoxia/reoxygenation-treated cardiomyocytes. Autophagy.

[9] Chokkalla A.K., Mehta S.L., Kim T., Chelluboina B., Kim J., Vemuganti R. (2019). Transient Focal Ischemia Significantly Alters the m(6) A Epitranscriptomic Tagging of RNAs in the Brain. Stroke.

[10] He P.C., He C. (2021). m(6) A RNA methylation: From mechanisms to therapeutic potential. EMBO J..

[11] Das Mandal S., Ray P.S. (2021). Transcriptome-wide analysis reveals spatial correlation between *N*^6^-methyladenosine and binding sites of microRNAs and RNA-binding proteins. Genomics.

[12] Di Timoteo G., Dattilo D., Centrón-Broco A., Colantoni A., Guarnacci M., Rossi F., Incarnato D., Oliviero S., Fatica A., Morlando M. (2020). Modulation of circRNA Metabolism by m^6^A Modification. Cell Rep..

[13] Yang D., Qiao J., Wang G., Lan Y., Li G., Guo X., Xi J., Ye D., Zhu S., Chen W. (2018). *N*^6^-Methyladenosine modification of lincRNA 1281 is critically required for mESC differentiation potential. Nucleic Acids Res..

[14] Zheng H.-X., Zhang X.-S., Sui N. (2020). Advances in the profiling of *N*^6^-methyladenosine (m^6^A) modifications. Biotechnol. Adv..

[15] Zhao B.S., Roundtree I.A., He C. (2017). Post-transcriptional gene regulation by mRNA modifications. Nat. Rev. Mol. Cell Biol..

[16] Sun T., Wu R., Ming L. (2019). The role of m^6^A RNA methylation in cancer. Biomed. Pharmacother..

[17] Xu Z., Lv B., Qin Y., Zhang B. (2022). Emerging Roles and Mechanism of m^6^A Methylation in Cardiometabolic Diseases. Cells.

[18] Desrosiers R., Friderici K., Rottman F. (1974). Identification of Methylated Nucleosides in Messenger RNA from Novikoff Hepatoma Cells. Proc. Natl. Acad. Sci. USA.

[19] Yue Y., Liu J., He C. (2015). RNA *N*^6^-methyladenosine methylation in post-transcriptional gene expression regulation. Genes Dev..

[20] Meyer K.D., Saletore Y., Zumbo P., Elemento O., Mason C.E., Jaffrey S.R. (2012). Comprehensive Analysis of mRNA Methylation Reveals Enrichment in 3′ UTRs and near Stop Codons. Cell.

[21] Dominissini D., Moshitch-Moshkovitz S., Schwartz S., Salmon-Divon M., Ungar L., Osenberg S., Cesarkas K., Jacob-Hirsch J., Amariglio N., Kupiec M. (2012). Topology of the human and mouse m^6^A RNA methylomes revealed by m^6^A-seq. Nature.

[22] Jia G., Fu Y., Zhao X., Dai Q., Zheng G., Yang Y., Yi C., Lindahl T., Pan T., Yang Y.-G. (2011). *N*^6^-Methyladenosine in nuclear RNA is a major substrate of the obesity-associated FTO. Nat. Chem. Biol..

[23] Chen T., Hao Y.-J., Zhang Y., Li M.-M., Wang M., Han W., Wu Y., Lv Y., Hao J., Wang L. (2015). m^6^A RNA Methylation Is Regulated by MicroRNAs and Promotes Reprogramming to Pluripotency. Cell Stem Cell.

[24] Vu L.P., Pickering B.F., Cheng Y., Zaccara S., Nguyen D., Minuesa G., Chou T., Chow A., Saletore Y., Mackay M. (2017). The *N*^6^-methyladenosine (m^6^A)-forming enzyme METTL3 controls myeloid differentiation of normal hematopoietic and leukemia cells. Nat. Med..

[25] Aoyama T., Yamashita S., Tomita K. (2020). Mechanistic insights into m^6^A modification of U6 snRNA by human METTL16. Nucleic Acids Res..

[26] Alderman M.H., Xiao A.Z. (2019). *N*^6^-Methyladenine in eukaryotes. Cell. Mol. Life Sci..

[27] Zhu D., Zhou J., Zhao J., Jiang G., Zhang X., Zhang Y., Dong M. (2019). ZC3H13 suppresses colorectal cancer proliferation and invasion via inactivating Ras–ERK signaling. J. Cell. Physiol..

[28] Patil D.P., Chen C.-K., Pickering B.F., Chow A., Jackson C., Guttman M., Jaffrey S.R. (2016). m^6^A RNA methylation promotes XIST-mediated transcriptional repression. Nature.

[29] Bokar J.A., Shambaugh M.E., Polayes D., Matera A.G., Rottman F.M. (1997). Purification and cDNA cloning of the AdoMet-binding subunit of the human mRNA (N6-adenosine)-methyltransferase. RNA.

[30] Wang P., Doxtader K.A., Nam Y. (2016). Structural Basis for Cooperative Function of Mettl3 and Mettl14 Methyltransferases. Mol. Cell.

[31] Koh C.W.Q., Goh Y.T., Goh W.S.S. (2019). Atlas of quantitative single-base-resolution N(6)-methyl-adenine methylomes. Nat. Commun..

[32] Liu J., Yue Y., Han D., Wang X., Fu Y., Zhang L., Jia G., Yu M., Lu Z., Deng X. (2014). A METTL3–METTL14 complex mediates mammalian nuclear RNA N6-adenosine methylation. Nat. Chem. Biol..

[33] Frayling T.M., Timpson N.J., Weedon M.N., Zeggini E., Freathy R.M., Lindgren C.M., Perry J.R., Elliott K.S., Lango H., Rayner N.W. (2007). A Common Variant in the FTO Gene Is Associated with Body Mass Index and Predisposes to Childhood and Adult Obesity. Science.

[34] Zheng G., Dahl J.A., Niu Y., Fedorcsak P., Huang C.-M., Li C.J., Vågbø C.B., Shi Y., Wang W.-L., Song S.-H. (2013). ALKBH5 Is a Mammalian RNA Demethylase that Impacts RNA Metabolism and Mouse Fertility. Mol. Cell.

[35] Yang J., Loos R.J.F., Powell J.E., Medland S.E., Speliotes E.K., Chasman D.I., Rose L.M., Thorleifsson G., Steinthorsdottir V., Mägi R. (2012). FTO genotype is associated with phenotypic variability of body mass index. Nature.

[36] Shah A., Rashid F., Awan H.M., Hu S., Wang X., Chen L., Shan G. (2017). The DEAD-Box RNA Helicase DDX3 Interacts with m^6^A RNA Demethylase ALKBH5. Stem Cells Int..

[37] Li H., Wu H., Wang Q., Ning S., Xu S., Pang D. (2021). Dual effects of *N*^6^-methyladenosine on cancer progression and immunotherapy. Mol. Ther. Nucleic Acids.

[38] Shi H., Wang X., Lu Z., Zhao B.S., Ma H., Hsu P.J., Liu C., He C. (2017). YTHDF3 facilitates translation and decay of *N*^6^-methyladenosine-modified RNA. Cell Res..

[39] Huang H., Weng H., Sun W., Qin X., Shi H., Wu H., Zhao B.S., Mesquita A., Liu C., Yuan C.L. (2018). Recognition of RNA *N*^6^-methyladenosine by IGF2BP proteins enhances mRNA stability and translation. Nat. Cell Biol..

[40] Geuens T., Bouhy D., Timmerman V. (2016). The hnRNP family: Insights into their role in health and disease. Hum. Genet..

[41] Neeland I.J., Ross R., Després J.-P., Matsuzawa Y., Yamashita S., Shai I., Seidell J., Magni P., Santos R.D., Arsenault B. (2019). Visceral and ectopic fat, atherosclerosis, and cardiometabolic disease: A position statement. Lancet Diabetes Endocrinol..

[42] Wu L., Pei Y., Zhu Y., Jiang M., Wang C., Cui W., Zhang D. (2019). Association of *N*^6^-methyladenine DNA with plaque progression in atherosclerosis via myocardial infarction-associated transcripts. Cell Death Dis..

[43] Lin L., Hales C.M., Garber K., Jin P. (2014). Fat mass and obesity-associated (FTO) protein interacts with CaMKII and modulates the activity of CREB signaling pathway. Hum. Mol. Genet..

[44] de Araujo T.M., Velloso L.A. (2020). Hypothalamic IRX3: A New Player in the Development of Obesity. Trends Endocrinol. Metab..

[45] Gleissner C.A., Leitinger N., Ley K. (2007). Effects of Native and Modified Low-Density Lipoproteins on Monocyte Recruitment in Atherosclerosis. Hypertension.

[46] Mo C., Yang M., Han X., Li J., Gao G., Tai H., Huang N., Xiao H. (2017). Fat mass and obesity-associated protein attenuates lipid accumulation in macrophage foam cells and alleviates atherosclerosis in apolipoprotein E-deficient mice. J. Hypertens..

[47] Wu Y.R., Shi X.Y., Ma C.Y., Zhang Y., Xu R.X., Li J.J. (2019). Liraglutide improves lipid metabolism by enhancing cholesterol efflux associated with ABCA1 and ERK1/2 pathway. Cardiovasc. Diabetol..

[48] Li D., Wang D., Wang Y., Ling W., Feng X., Xia M. (2010). Adenosine Monophosphate-activated Protein Kinase Induces Cholesterol Efflux from Macrophage-derived Foam Cells and Alleviates Atherosclerosis in Apolipoprotein E-deficient Mice. J. Biol. Chem..

[49] Liang M. (2018). Epigenetic Mechanisms and Hypertension. Hypertension.

[50] Wu Q., Yuan X., Han R., Zhang H., Xiu R. (2019). Epitranscriptomic mechanisms of *N*^6^-methyladenosine methylation regulating mammalian hypertension development by determined spontaneously hypertensive rats pericytes. Epigenomics.

[51] Zheng Y., Nie P., Peng D., He Z., Liu M., Xie Y., Miao Y., Zuo Z., Ren J. (2018). m^6^AVar: A database of functional variants involved in m^6^A modification. Nucleic Acids Res..

[52] Mo X.-B., Lei S.-F., Zhang Y.-H., Zhang H. (2019). Examination of the associations between m^6^A-associated single-nucleotide polymorphisms and blood pressure. Hypertens. Res..

[53] Scott L.J., Mohlke K.L., Bonnycastle L.L., Willer C.J., Li Y., Duren W.L., Erdos M.R., Stringham H.M., Chines P.S., Jackson A.U. (2007). A Genome-Wide Association Study of Type 2 Diabetes in Finns Detects Multiple Susceptibility Variants. Science.

[54] Äijälä M., Ronkainen J., Huusko T., Malo E., Savolainen E.-R., Savolainen M.J., Salonurmi T., Bloigu R., Kesäniemi Y.A., Ukkola O. (2015). The fat mass and obesity-associated (FTO) gene variant rs9939609 predicts long-term incidence of cardiovascular disease and related death independent of the traditional risk factors. Ann. Med..

[55] Yang Y., Shen F., Huang W., Qin S., Huang J.-T., Sergi C., Yuan B.-F., Liu S.-M. (2019). Glucose Is Involved in the Dynamic Regulation of m^6^A in Patients With Type 2 Diabetes. J. Clin. Endocrinol. Metab..

[56] De Jesus D.F., Zhang Z., Kahraman S., Brown N.K., Chen M., Hu J., Gupta M.K., He C., Kulkarni R.N. (2019). m^6^A mRNA Methylation Regulates Human beta-Cell Biology in Physiological States and in Type 2 Diabetes. Nat. Metab..

[57] Chatzizisis Y.S., Coskun A.U., Jonas M., Edelman E.R., Feldman C.L., Stone P.H. (2007). Role of Endothelial Shear Stress in the Natural History of Coronary Atherosclerosis and Vascular Remodeling: Molecular, Cellular, and Vascular Behavior. J. Am. Coll. Cardiol..

[58] Zhu W., Zhang H., Wang S. (2022). Vitamin D3 Suppresses Human Cytomegalovirus-Induced Vascular Endothelial Apoptosis via Rectification of Paradoxical m^6^A Modification of Mitochondrial Calcium Uniporter mRNA, Which Is Regulated by METTL3 and YTHDF3. Front. Microbiol..

[59] Wang L.J., Xue Y., Li H., Huo R., Yan Z., Wang J., Xu H., Wang J., Cao Y., Zhao J.Z. (2020). Wilms’ tumour 1-associating protein inhibits endothelial cell angiogenesis by m^6^A-dependent epigenetic silencing of desmoplakin in brain arteriovenous malformation. J. Cell. Mol. Med..

[60] Kumari R., Dutta R., Ranjan P., Suleiman Z.G., Goswami S.K., Li J., Pal H.C., Verma S.K. (2021). ALKBH5 Regulates SPHK1-Dependent Endothelial Cell Angiogenesis Following Ischemic Stress. Front. Cardiovasc. Med..

[61] Li J., Meng Q., Fu Y., Yu X., Ji T., Chao Y., Chen Q., Li Y., Bian H. (2021). Novel insights: Dynamic foam cells derived from the macrophage in atherosclerosis. J. Cell. Physiol..

[62] Zhao W., Wang Z., Sun Z., He Y., Jian D., Hu X., Zhang W., Zheng L. (2018). RNA helicase DDX5 participates in oxLDL-induced macrophage scavenger receptor 1 expression by suppressing mRNA degradation. Exp. Cell Res..

[63] Park M.H., Jeong E., Choudhury M. (2020). Mono-(2-Ethylhexyl)phthalate Regulates Cholesterol Efflux via MicroRNAs Regulated m^6^A RNA Methylation. Chem. Res. Toxicol..

[64] Ketelhuth D.F.J., Lutgens E., Bäck M., Binder C.J., Van den Bossche J., Daniel C., Dumitriu I.E., Hoefer I., Libby P., O’Neill L. (2019). Immunometabolism and atherosclerosis: Perspectives and clinical significance: A position paper from the Working Group on Atherosclerosis and Vascular Biology of the European Society of Cardiology. Cardiovasc. Res..

[65] Liu Y., Liu Z., Tang H., Shen Y., Gong Z., Xie N., Zhang X., Wang W., Kong W., Zhou Y. (2019). The *N*^6^-Methyladenosine (m^6^A)-Forming Enzyme METTL3 Facilitates M1 Macrophage Polarization through the Methylation of STAT1 mRNA. Am. J. Physiol. Cell. Physiol..

[66] Huangfu N., Zheng W., Xu Z., Wang S., Wang Y., Cheng J., Li Z., Cheng K., Zhang S., Chen X. (2020). RBM4 regulates M1 macrophages polarization through targeting STAT1-mediated glycolysis. Int. Immunopharmacol..

[67] Gu X., Zhang Y., Li D., Cai H., Cai L., Xu Q. (2020). *N*^6^-methyladenosine demethylase FTO promotes M1 and M2 macrophage activation. Cell. Signal..

[68] Li Z., Xu Q., Huangfu N., Chen X., Zhu J. (2022). Mettl3 promotes oxLDL-mediated inflammation through activating STAT1 signaling. J. Clin. Lab. Anal..

[69] Wang J., Yan S., Lu H., Wang S., Xu D. (2019). METTL3 Attenuates LPS-Induced Inflammatory Response in Macrophages via NF-kappaB Signaling Pathway. Mediat. Inflamm..

[70] Yu R., Li Q., Feng Z., Cai L., Xu Q. (2019). m^6^A Reader YTHDF2 Regulates LPS-Induced Inflammatory Response. Int. J. Mol. Sci..

[71] Yu Z., Zheng X., Wang C., Chen C., Ning N., Peng D., Liu T., Pan W. (2022). The Traditional Chinese Medicine Hua Tuo Zai Zao Wan Alleviates Atherosclerosis by Deactivation of Inflammatory Macrophages. Evid.-Based Complement. Altern. Med..

[72] Zheng Y., Li Y., Ran X., Wang D., Zheng X., Zhang M., Yu B., Sun Y., Wu J. (2022). Mettl14 mediates the inflammatory response of macrophages in atherosclerosis through the NF-kappaB/IL-6 signaling pathway. Cell. Mol. Life Sci..

[73] Zhang X., Li X., Jia H., An G., Ni J. (2021). The m^6^A methyltransferase METTL3 modifies PGC-1alpha mRNA promoting mitochondrial dysfunction and oxLDL-induced inflammation in monocytes. J. Biol. Chem..

[74] Guo M., Yan R., Ji Q., Yao H., Sun M., Duan L., Xue Z., Jia Y. (2020). IFN regulatory Factor-1 induced macrophage pyroptosis by modulating m^6^A modification of circ_0029589 in patients with acute coronary syndrome. Int. Immunopharmacol..

[75] Bennett M.R., Sinha S., Owens G.K. (2016). Vascular Smooth Muscle Cells in Atherosclerosis. Circ. Res..

[76] Lin J., Zhu Q., Huang J., Cai R., Kuang Y. (2020). Hypoxia Promotes Vascular Smooth Muscle Cell (VSMC) Differentiation of Adipose-Derived Stem Cell (ADSC) by Regulating Mettl3 and Paracrine Factors. Stem Cells Int..

[77] Chen J., Ning Y., Zhang H., Song N., Gu Y., Shi Y., Cai J., Ding X., Zhang X. (2019). METTL14-dependent m^6^A regulates vascular calcification induced by indoxyl sulfate. Life Sci..

[78] Zhu B., Gong Y., Shen L., Li J., Han J., Song B., Hu L., Wang Q., Wang Z. (2020). Total Panax notoginseng saponin inhibits vascular smooth muscle cell proliferation and migration and intimal hyperplasia by regulating WTAP/p16 signals via m^6^A modulation. Biomed. Pharmacother..

[79] Ma D., Liu X., Zhang J.J., Zhao J.J., Xiong Y.J., Chang Q., Wang H.Y., Su P., Meng J., Zhao Y.-B. (2020). Vascular Smooth Muscle FTO Promotes Aortic Dissecting Aneurysms via m^6^A Modification of Klf5. Front. Cardiovasc. Med..

[80] Huo Y.B., Gao X., Peng Q., Nie Q., Bi W. (2022). Dihydroartemisinin alleviates AngII-induced vascular smooth muscle cell proliferation and inflammatory response by blocking the FTO/NR4A3 axis. Inflamm. Res..

[81] Deng K., Ning X., Ren X., Yang B., Li J., Cao J., Chen J., Lu X., Chen S., Wang L. (2021). Transcriptome-wide *N*^6^-methyladenosine methylation landscape of coronary artery disease. Epigenomics.

[82] Yuan J., Liu Y., Zhou L., Xue Y., Lu Z., Gan J. (2021). YTHDC2-Mediated circYTHDC2 *N*^6^-Methyladenosine Modification Promotes Vascular Smooth Muscle Cells Dysfunction Through Inhibiting Ten-Eleven Translocation 2. Front. Cardiovasc. Med..

[83] Zhang B.-F., Wu Z.H., Deng J., Jin H.J., Chen W.B., Zhang S., Liu X.J., Wang W.-T., Zheng X.-T. (2022). m^6^A methylation-mediated elevation of SM22α inhibits the proliferation and migration of vascular smooth muscle cells and ameliorates intimal hyperplasia in type 2 diabetes mellitus. Biol. Chem..

[84] Yao M.-D., Jiang Q., Ma Y., Liu C., Zhu C.-Y., Sun Y.-N., Shan K., Ge H.-M., Zhang Q.-Y., Zhang H.-Y. (2020). Role of METTL3-Dependent *N*^6^-Methyladenosine mRNA Modification in the Promotion of Angiogenesis. Mol. Ther..

[85] Dong G., Yu J., Shan G., Su L., Yu N., Yang S. (2021). *N*^6^-Methyladenosine Methyltransferase METTL3 Promotes Angiogenesis and Atherosclerosis by Upregulating the JAK2/STAT3 Pathway via m^6^A Reader IGF2BP1. Front. Cell. Dev. Biol..

[86] Chien C.-S., Li J.Y.-S., Chien Y., Wang M.-L., Yarmishyn A.A., Tsai P.-H., Juan C.-C., Nguyen P., Cheng H.-M., Huo T.-I. (2021). METTL3-dependent N ^6^ -methyladenosine RNA modification mediates the atherogenic inflammatory cascades in vascular endothelium. Proc. Natl. Acad. Sci. USA.

[87] Chen J., Lai K., Yong X., Yin H., Chen Z., Wang H., Chen K., Zheng J. (2022). Silencing METTL3 Stabilizes Atherosclerotic Plaques by Regulating the Phenotypic Transformation of Vascular Smooth Muscle Cells via the miR-375-3p/PDK1 Axis. Cardiovasc. Drugs Ther..

[88] Li B., Zhang T., Liu M., Cui Z., Zhang Y., Liu M., Liu Y., Sun Y., Li M., Tian Y. (2021). RNA *N*^6^-methyladenosine modulates endothelial atherogenic responses to disturbed flow in mice. eLife.

[89] Jian D., Wang Y., Jian L., Tang H., Rao L., Chen K., Jia Z., Zhang W., Liu Y., Chen X. (2020). METTL14 aggravates endothelial inflammation and atherosclerosis by increasing FOXO1 *N*^6^-methyladeosine modifications. Theranostics.

[90] Zhang B.Y., Han L., Tang Y.F., Zhang G.X., Fan X.-L., Zhang J.J., Xue Q., Xu Z.Y. (2022). METTL14 regulates m^6^A methylation-modified primary miR-19a to promote cardiovascular endothelial cell proliferation and invasion. Eur. Rev. Med. Pharmacol. Sci..

[91] Tang X., Yin R., Shi H., Wang X., Shen D., Wang X., Pan C. (2020). LncRNA ZFAS1 confers inflammatory responses and reduces cholesterol efflux in atherosclerosis through regulating miR-654-3p-ADAM10/RAB22A axis. Int. J. Cardiol..

[92] Liu Y., Luo G., Tang Q., Song Y., Liu D., Wang H., Ma J. (2022). Methyltransferase-like 14 silencing relieves the development of atherosclerosis via m^6^A modification of p65 mRNA. Bioengineered.

[93] Wang J., Zhang J., Ma Y., Zeng Y., Lu C., Yang F., Jiang N., Zhang X., Wang Y., Xu Y. (2021). WTAP promotes myocardial ischemia/reperfusion injury by increasing endoplasmic reticulum stress via regulating m^6^A modification of ATF4 mRNA. Aging.

[94] Mathiyalagan P., Adamiak M., Mayourian J., Sassi Y., Liang Y., Agarwal N., Jha D., Zhang S., Kohlbrenner E., Chepurko E. (2019). FTO-Dependent m^6^A Regulates Cardiac Function During Remodeling and Repair. Circulation.

[95] Shen W., Li H., Su H., Chen K., Yan J. (2021). FTO overexpression inhibits apoptosis of hypoxia/reoxygenation-treated myocardial cells by regulating m^6^A modification of Mhrt. Mol. Cell. Biochem..

[96] Zhao Y., Hu J., Sun X., Yang K., Yang L., Kong L., Zhang B., Li F., Li C., Shi B. (2021). Loss of m^6^A demethylase ALKBH5 promotes post-ischemic angiogenesis via post-transcriptional stabilization of WNT5A. Clin. Transl. Med..

[97] Si W., Li Y., Ye S., Li Z., Liu Y., Kuang W., Chen D., Zhu M. (2020). Methyltransferase 3 Mediated miRNA m^6^A Methylation Promotes Stress Granule Formation in the Early Stage of Acute Ischemic Stroke. Front. Mol. Neurosci..

[98] Zhang Z., Wang Q., Zhao X., Shao L., Liu G., Zheng X., Xie L., Zhang Y., Sun C., Xu R. (2020). YTHDC1 mitigates ischemic stroke by promoting Akt phosphorylation through destabilizing PTEN mRNA. Cell Death Dis..

[99] Benjamin E.J., Virani S.S., Callaway C.W., Chamberlain A.M., Chang A.R., Cheng S., Chiuve S.E., Cushman M., Delling F.N., Deo R. (2018). Heart Disease and Stroke Statistics-2018 Update: A Report From the American Heart Association. Circulation.

[100] Chen L., Yao H., Hui J.-y., Ding S.-h., Fan Y.-l., Pan Y.-h., Chen K.-h., Wan J.-q., Jiang J.-y. (2016). Global transcriptomic study of atherosclerosis development in rats. Gene.

[101] Mahmoudi M., Yu M., Serpooshan V., Wu J.C., Langer R., Lee R.T., Karp J.M., Farokhzad O.C. (2017). Multiscale technologies for treatment of ischemic cardiomyopathy. Nat. Nanotechnol..

[102] Diao M.Y., Zhu Y., Yang J., Xi S.S., Wen X., Gu Q., Hu W. (2020). Hypothermia protects neurons against ischemia/reperfusion-induced pyroptosis via m^6^A-mediated activation of PTEN and the PI3K/Akt/GSK-3beta signaling pathway. Brain Res. Bull..

[103] Liu Q., Li J., Hartstone-Rose A., Wang J., Li J., Janicki J.S., Fan D. (2015). Chinese Herbal Compounds for the Prevention and Treatment of Atherosclerosis: Experimental Evidence and Mechanisms. Evid.-Based Complement. Altern. Med..

[104] Shen D.-Z., Xin S.-L., Chen C., Liu T. (2013). Effect of atorvastatin on expression of TLR4 and NF-κB p65 in atherosclerotic rabbits. Asian Pac. J. Trop. Med..

